# A cross-sectional study of the relationship between injuries and quality of life, psychological distress, sleeping problems, and global subjective health in adults from three Norwegian counties

**DOI:** 10.1186/s12955-023-02191-1

**Published:** 2023-11-03

**Authors:** Leif Edvard Aarø, Eyvind Ohm, Jens Christoffer Skogen, Thomas Nilsen, Marit Knapstad, Øystein Vedaa, Ragnhild Bang Nes, Benjamin Clarsen, Knut-Inge Klepp

**Affiliations:** 1 Department of Health Promotion, Norwegian Institute of Public Health, Zander Kaaesgt. 7, NO-5015 Bergen, Norway; 2 Department of Health and Inequality, Norwegian Institute of Public Health, Marcus Thranes Gate 6, NO-0473 Oslo, Norway; 3 Centre for Alcohol & Drug Research, Stavanger University Hospital, Lagårdsveien 78, NO-4068 Stavanger, Norway; 4 Department of Mental Health and Suicide, Norwegian Institute of Public Health, Sandakerveien 24C, NO-0473 Oslo, Norway; 5 Division of Mental and Physical Health, Norwegian Institute of Public Health, Marcus Thranes Gate 6, NO-0473 Oslo, Norway

**Keywords:** Injuries, Quality of life, Distress, Sleeping problems, Global subjective health, Population survey, Cross-sectional, Adults

## Abstract

**Background:**

Studies examining associations between injuries and outcomes like quality of life and psychological distress are important to understand a broader range of possible consequences of injuries for population health.

**Aims:**

The aim of this study was to examine associations between self-reported injury and quality of life, psychological distress, sleeping problems, and global subjective health.

**Methods:**

The sample was drawn from the Norwegian National Population Register. Data were collected among the general adult populations in three Norwegian counties in 2019–2020 (response rate 45.3%, *n* = 74,030). Exposure variables were being injured during the last 12 months, cause of injury (if more than one, the most serious one), and functional impairment due to injuries. Outcome variables included measures of total quality of life, global quality of life, positive affect, negative affect, positive social relations, social capital (trust, belongingness, feeling safe), psychological distress, sleep problems, loneliness, and global subjective health. Data were analysed with General Linear Modelling in SPSS Complex.

**Results:**

Reporting to have been injured once during the last 12 months was associated with slightly elevated levels of psychological distress, sleeping problems, and loneliness, and lower mean scores on quality-of-life indicators and global subjective health. Reporting being injured twice or more showed more pronounced contrasts to the reference group on the same outcomes, with Cohen’s *d*-values (absolute numbers) ranging from 0.17 to 0.54. For having been victim to violence, *d*-values ranged from 0.30 to 1.01. Moderate functional impairment due to injuries was associated with less favourable scores on all outcomes (*d* ranging from 0.15 to 0.71). For strong functional impairment *d*-values ranged from 0.35 to 1.17.

**Conclusions:**

Elevated levels of distress and reduced levels of quality of life are particularly associated with multiple injuries, being victim to violence, and functional impairment due to injuries. Prospective, longitudinal studies with high quality instruments and large samples, allowing adjustment for baseline values of outcome variables, and utilization of state-of-the-art statistical techniques, would bring this research closer to examining causality.

## Introduction

Worldwide, injuries such as those caused by road traffic crashes, falls, interpersonal violence and self-harm represent a serious public health challenge [1]. Although the burdens represented by injuries are declining in Norway, in 2015, 5.5% of all deaths were caused by injuries, and injuries caused 9.5% of years lost [2]. In terms of Disability Adjusted Life Years (DALYs), the loss from injuries amounted to 7.6% [2].

Injuries are usually painful for the individual, impose burdens on health- and welfare services, and lead to loss of productive worktime [3]. These are the immediate (often also long-term), and most tangible costs. Injuries may, however, also have consequences in terms of increased levels of psychological distress, reduced social functioning, and reduced wellbeing. This kind of possible effects of injuries are addressed in the present study.

Many studies of the relationship between being injured and aspects of distress and quality of life have focussed on patients with specific diagnoses. Serious limitation of daily functioning, high levels of distress, and low levels of health-related quality of life have been found in groups of patients, for instance after traumatic brain injuries [4], hip fractures [5], and traumatic pelvic injuries [6].

Studies among broader groups of injured patients confirm these findings [7–23]. Post-injury scores are often compared with pre-injury scores obtained retrospectively during post-trauma interviews. Levels of distress have been shown to be higher and levels of subjective quality of life to be lower after injuries, and multiple injuries are associated with stronger associations with outcomes (dose–response) [7–13]. Prospective, longitudinal studies among patients being treated for injuries in health care settings have showed improvements in scores as a function of time after the first post-injury data collection [11, 14, 18–20, 24–26], although also other patterns of change have been observed [27–29].

A serious methodological challenge in studies where post-injury scores are compared with pre-injury scores obtained retrospectively, is the retrospective measurement of pre-injury levels of distress and quality of life. Retrospective measurement is based on the assumption that scores are similar to those that would have been obtained with actual measurements before injury. This is not necessarily the case[30].

In other studies, distress and subjective quality of life scores among injured people are compared with normative data. Higher levels of distress and lower levels of subjective quality of life are found among the injured [7, 14–23]. Scores less favourable than in normative data have been found long after the injury took place, after 12 months [11, 12, 15, 17, 22], 24 months, [9, 15, 19, 31], and after five to six years [20, 23]. Serious challenges in these studies are to find data from sufficiently relevant normative populations and data collections taking place sufficiently close in time to the data collections among injured.

In a recent systematic review of twenty-nine studies, it was found that being injured was associated with lower health-related quality of life compared to not being injured. Most studies documented improvements in health-related quality of life over time since shortly after the injury event, but without full return to pre-injury levels [32]. Improvements tend to be fastest during the first period after injury, and less fast or not at all during subsequent months.

There is a large literature on consequences of injuries which is beyond the scope of this study to cover. One example is a study by Andelic and associates, which describes disability and quality of life 20 years after traumatic brain injury [33].

Exposure to violence is associated with deteriorations in health and well-being [34–36]. Studies of consequences of intimate partner violence among women have shown exposure to violence to be associated with long-term negative psychological effects and increased risks of suicide [37]. Effects of violence on outcomes such as health is well documented [36].

In a study among women in Finland, it was found that exposure to violence in close relationships was associated with lower mean scores on quality of life [34]. Two Danish cross-sectional studies provided evidence for an association between exposure to physical violence and reduction in health-related quality of life [35].

Abused women in Norway were in one study found to have lower scores than national normative data on all dimensions of the SF-36 scale (physical health, role-physical, bodily pain, general health, vitality, social functioning, role-emotional, mental health) at a first data collection (while staying in women’s shelter). One year later, mean scores had improved markedly, except for vitality scores [16].

Studies of effects of violence are, however, not the same as studies of effects of injuries caused by violence. In a study from Australia, it was reported that adults exposed to physical violence and serious injury exhibited lower levels of health-related quality of life. Exposure to injuries was measured independently from exposure to violence. The injuries reported were therefore, however, not necessarily caused by violence [38].

Studies with relevant designs and sufficient power on associations between functional impairment due to injuries and distress and quality of life indicators were not found.

The purpose of the present study was to examine associations between a set of three injury-related predictors and a selection of outcome variables related to distress and subjective quality of life.

## Methods

### Data collections

Starting in 2018, large-scale questionnaire-based data collections on a variety of topics relevant for public health action, including data on injuries, distress, and quality of life, have been carried out in counties all over Norway (The Norwegian Counties Public Health Survey) [39]. This study is based on data from three Norwegian counties (Agder, Nordland, and Troms & Finnmark). Samples were drawn from the Norwegian National Population Register and “washed” against the Common Contact Register to exclude those who had reserved against participation in surveys and to retrieve digital contact information. The total number of people invited was 163 817. Participation rate was 45.2% (*n* = 74,030). The Norwegian Population Register contains data on all individuals currently residing in or who have previously resided in Norway. The register forms the basis for the tax register, the electoral register and population statistics. Important purposes of the population register are to ensure that all citizens receive information from public authorities and that their rights and obligations are safeguarded [40].

Internet-based data collections were carried out by the Norwegian Institute of Public Health. Study participants could respond to the questionnaire by smartphones, tablets, or computers. Data collections (two reminders) were carried out in May – June 2019 in Troms & Finnmark, January – February 2020 in Nordland, and September – October 2020 in Agder. Reminders were sent only to those who had not responded.

### Instruments

The results presented in this manuscript are based on the following four questions about injuries and functional impairment:

- “During the last 12 months, have you experienced an injury which had to be treated by a medical doctor or dentist?” Response categories: 1 – “Yes, once”; 2 – “Twice or more”; 3 – “No”.
- If “Yes, once” or “Yes, twice or more” on the previous question: “What caused the injury? (If more than one injury, focus on the most serious one).” Response categories: 1 – “Accident”, 2 – “Violence/assault”, 3 – “Other cause”.
- “Do you have any functional impairment resulting from injury? Impairments which are not permanent but appear from time to time are included.” Response categories: 1—“Yes”; 2 – “No”. Injuries referred to in this question could have taken place at any time earlier in the life of the study participant and are not limited to those which took place during the 12 months prior to responding to the questionnaire.
- If “Yes” on previous question: How do these functional impairments influence your daily life? Response categories: 1—“To a large extent”; 2 – “To some extent”; 3 – “To a small extent”; 4 – “Not at all”.

To measure distress during the last week (7 days), we used a 5-item version of the Hopkins Symptom Checklist (HSCL-5) which includes items on depression and anxiety [32]. The items cover the following symptoms: (i) “Nervousness or shakiness inside”, (ii) “Feeling fearful”, (iii) “Feeling hopeless about the future”, (iv) “Feeling blue”, and (v) “Worrying too much about things”. Response categories were 1 – “Not at all” 2 – “A little”, 3 – “Quite a lot”, and 4 – “Very much”. Studies have confirmed good psychometric properties such as high correlations of sumscores with the 25-item version, high alpha values [41–43], and measurement invariance [43]. Strand and associates have suggested that scores higher than 2.00 should be considered as high [42].

The quality of life scales were first published in a report from the Norwegian Directorate of Health, [44] and in a more recent publication, their psychometric properties have been examined [45]. The measures include:

- Global quality of life (satisfaction with life, life being meaningful)
- Positive affect (happiness, involvement)
- Negative affect (being worried, feeling blue, being irritable, loneliness, anxiousness)
- Positive social relations (supportive social relations, contributes actively to others’ happiness and quality of life)
- Social capital (trust, belongingness, feeling safe)

The response format was scales from 0 to 10 with labels indicating the extremes, for instance, with regard to satisfaction with life: “Not satisfied at all” and “Most satisfied”.

Cronbach’s alpha values for the five groups of items were shown to vary from 0.64 to 0.88 and the five meanscores were highly correlated. A meanscore for “Total quality of life” could therefore be constructed [45].

Sleeping problems was measured with a single item: “To what extent have you been bothered by sleeping problems during the last week?”. Response categories were 1 – “Not bothered”; 2 – “A little”; 3 – “Quite a lot”, 4 – “Very much”.

Loneliness was measured with the short version of the UCLA loneliness scale [46, 47]: (i) “How often do you feel that you lack companionship”; (ii) “How often do you feel left out?”; (iii) “How often do you feel isolated from others?”. Response categories were: 1 – “Never”; 2 – “Seldom”; 3 – “Sometimes”; 4 – “Often”; and 5 - “Very often”. Cronbach’s alpha for this scale in the present data was 0.86.

Global subjective health was measured with one item: “How do you assess your own health? Would you say it is …”. And then a set of response categories: 1 – “Very good”; 2 – “Good”; 3 – “Neither good nor poor”; 4 – “Poor”; 5 – “Very poor” [48].

### Data analysis

All data analyses were carried out with the Complex module of SPSS version 27. Since a few municipalities were oversampled, the data from these municipalities were weighted down correspondingly. We used design weighting. Since the proportion sampled was doubled in the smallest municipalities, all participants from these municipalities received a weight of 0.50. The participants who were weighted down this way corresponded to 3.9 per cent of the total sample. General Linear Modelling was used for analysis of predictors on quality of life, psychological distress, sleeping problems, and global subjective health. In all GLM analyses (Shown in Tables 1, 2 and 3), adjustments are made for gender and age (categorized) defined as fixed effects categorical variables. Predictors in these tables are being injured (not injured; injured once; injured twice or more) during the last twelve months (Table 1), cause of injury (not injured; accident; victim of violence; other cause) (Table 2), and functional impairment due to injury (not injured; injured, but only small effects in daily life; injured, with some effects in daily life; injured, with strong effects in daily life) (Table 3). All three tables contain the same set of outcomes: total quality of life, global quality of life, positive affect, negative affect, social relations, social capital and three aspects of social capital (trust, belongingness, feeling safe), Hopkins Symptoms Checklist five items version, sleeping problems, loneliness, and global subjective health.

**Table 1:**
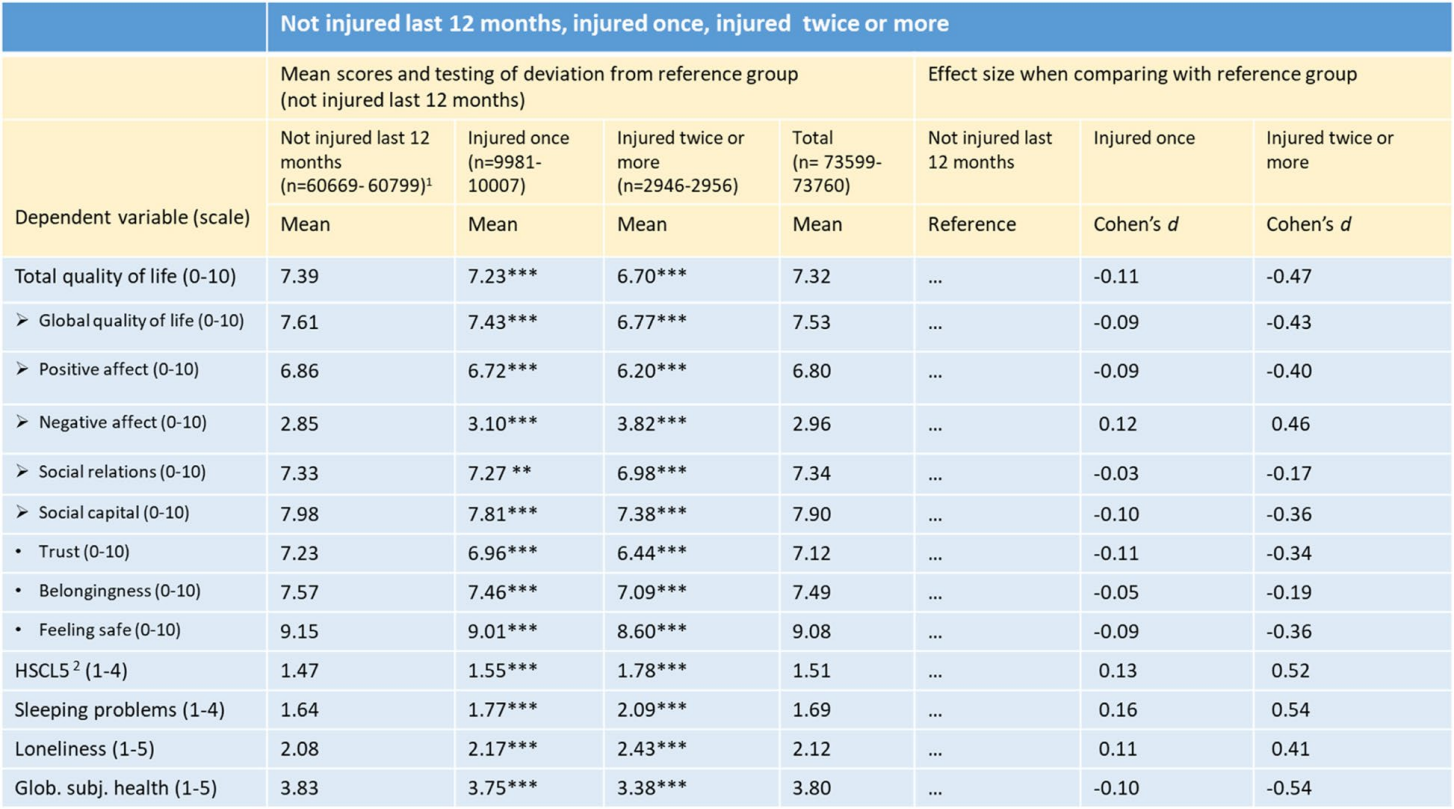
Psychological distress and quality of life indicators by being injured last 12 months adjusted for gender and age. Weighted for oversampling in specific municipalities. Analyses in SPSS Complex, General Linear Modelling

^1^Unweighted numbers. Since the number of missing is low and number of observations does not vary much, n is shown as intervals

^2^Hopkins Symptom Checklist, five-items version

**: *p* < .01; ***: *p* < .001

**Table 2:**
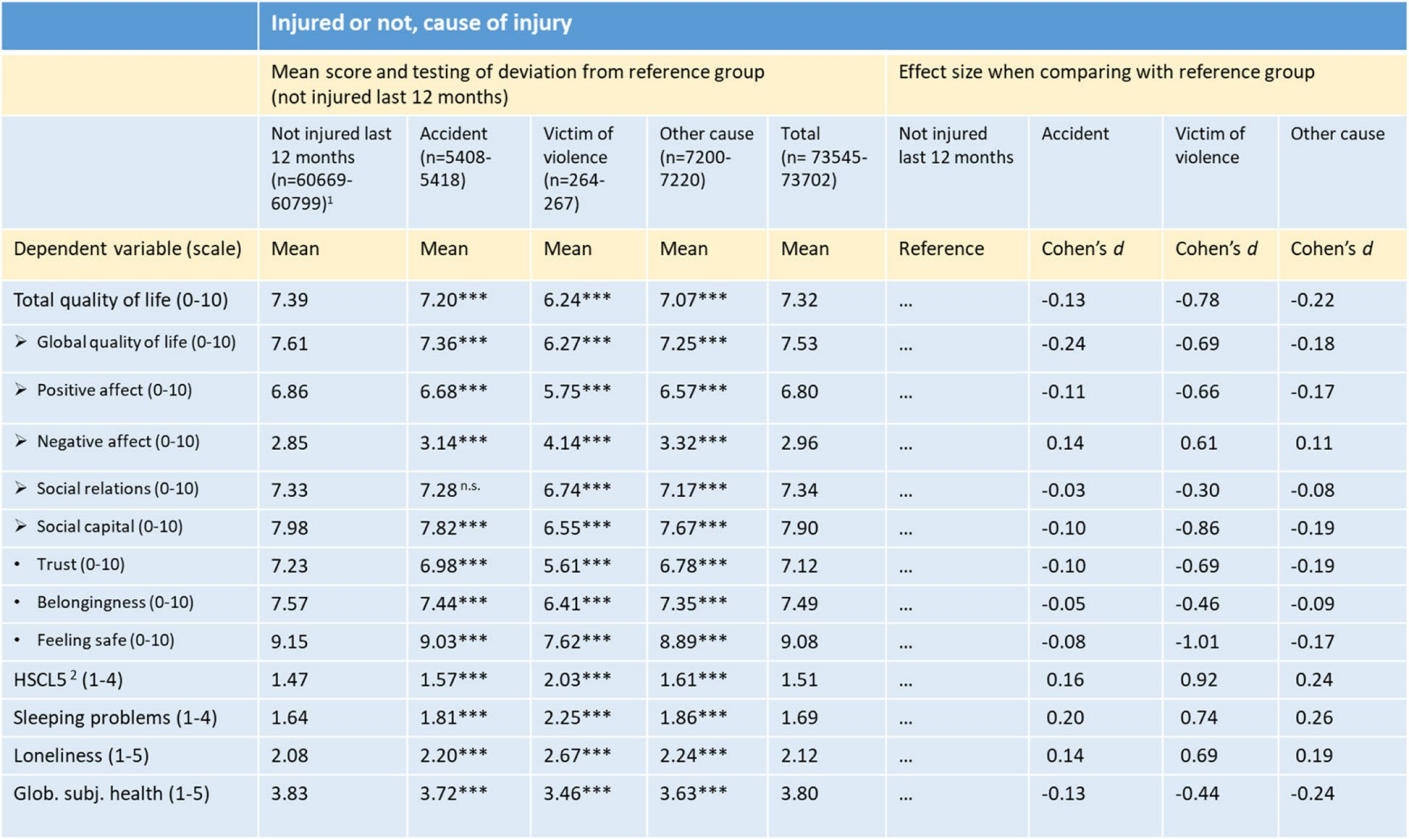
Psychological distress and quality of life indicators by being injured last 12 months and cause adjusted for gender and age. Weighted for oversampling in specific municipalities. Analyses in SPSS Complex, General Linear Modelling

^1^ Unweighted numbers. Since the number of missing is low and number of observations does not vary much, n is shown as intervals

^2^Hopkins Symptom Checklist, five-items version

***: *p* < .001; *n.s*. Not significant

**Table 3:**
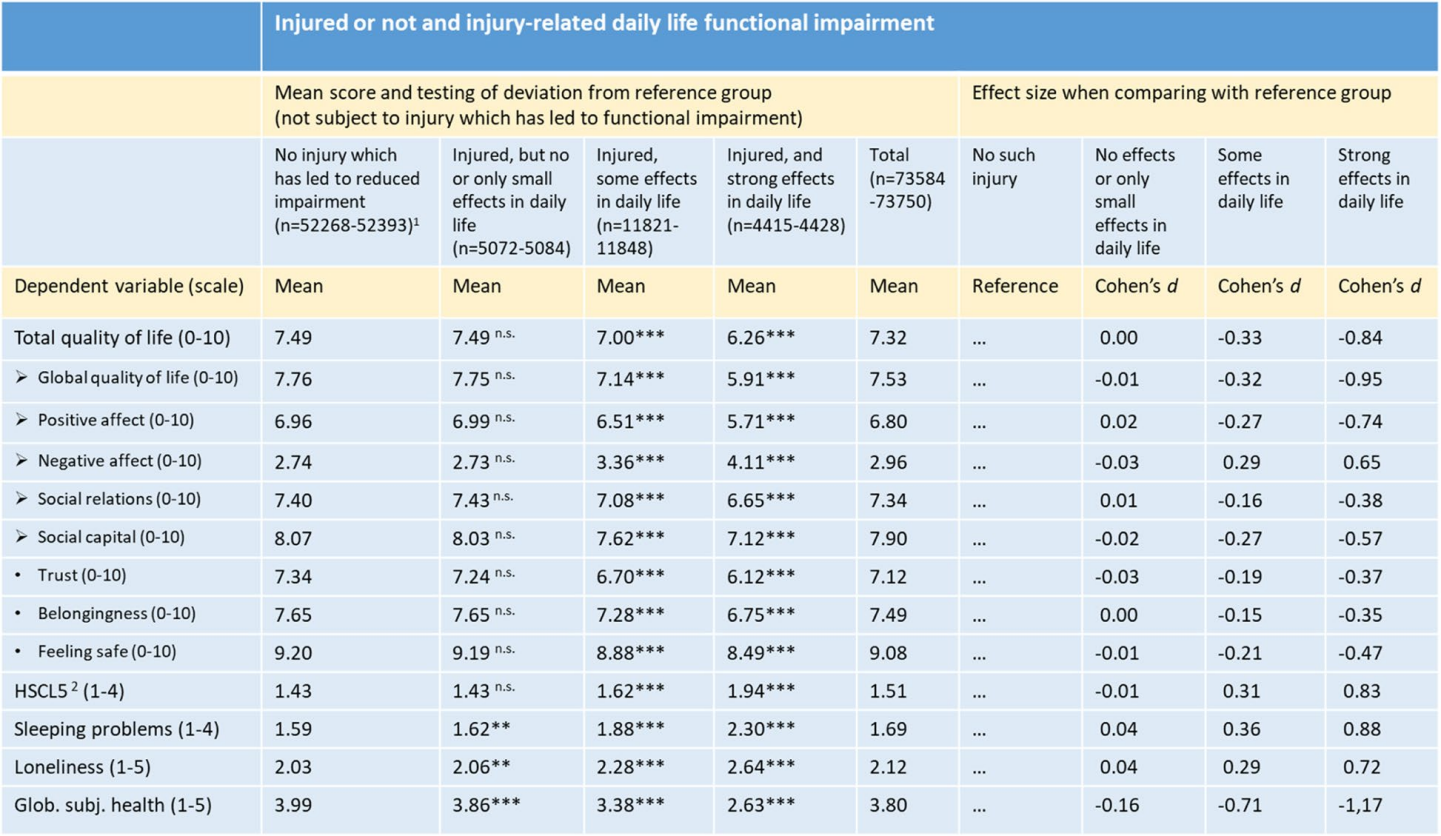
Psychological distress and quality of life indicators by functional impairment adjusted for gender and age. Weighted for oversampling in specific municipalities. Analyses in SPSS Complex, General Linear Models

^1^Unweighted numbers. Since the number of missing is low and number of observations does not vary much, n is shown as intervals.

^2^Hopkins Symptom Checklist, five-items version.

**: *p* < .01; ***: *p* < .001; n.s.: Not significant

In all tables, various groups of people who have reported to have been injured are compared with reference groups of people not injured. To make interpretation easier, all differences between the reference groups and the “exposed” groups are presented as Cohen’s *d* coefficients [49].

## Results

### Injury variables

The total number of study participants was 74,030, 53.7% women and 46.3% men. Mean age was 47.7 years, standard deviation was 16.2 years. Age range was 18 to 95 years.

The proportion who reported that they had been so seriously injured during the last 12 months that they had been in touch with a medical doctor or dentist was 17.6% (13.5% once and 4.0% twice or more). The proportions were 15.4% among women and 20.0% among men (F_ADJUSTED_ = 259.703; d.f. = 1 and 74,029; *p* < 0.001). The proportion who reported being injured at least once during the last 12 months varied by age. Among women, this proportion varied from 13.5% for those aged 60–69 years to 19.2% for those aged 18–29 years (F_ADJUSTED_ = 25.147; d.f. = 5 and 198,459.188; *p* < 0.001). Among men, this proportion varied between 17.4% (age 60–69) and 23.6% (age 18–29) (F_ADJUSTED_ = 16.810; d.f. = 5 and 171,679.795; *p* < 0.001).

Among those who reported to have been injured during the last 12 months, 2.1% reported that they had been victims of violence, 2.6% among women and 1.7% among men (F_ADJUSTED_ = 11.200; d.f. = 1 and 74,029; *p* < 0.001).

The proportion who reported that they had functional impairment due to injury (earlier in life, not limited to the last 12 months) was 28.9%, 28.4% among women and 29.5% among men (F_ADJUSTED_ = 11.221; d.f. = 1 and 74,029; *p* < 0.001).

Among those who reported to have functional impairment due to injury, 23.9% reported no or small degree of impact in daily life. Some degree of impact was reported by 55.4% and high degree by 20.7%. Larger proportions of women than men reported “Some degree of impact” (57.2% among women and 53.4% among men) or “High degree of impact” (22.0% among women and 19.3% among men (F_ADJUSTED_ = 62.420; d.f. = 2 and 148,057.938; *p* < 0.001).

### Outcome variables by injury-related variables

In Table 1, all outcome variables (Quality of life, Distress (HSCL-5), Sleeping-problems, Loneliness and Global subjective health) are analyzed against self-reports of not being injured, being injured once, or being injured twice or more during the last 12 months.

All contrasts between the “Injured once”-group and those reporting not to have been injured during the last 12 months were statistically significant, but small, generally ranging from *d* = 0.09 to 0.16. Only two outcome variables showed smaller contrasts, Belongingness (*d* = -0.05) and Positive social relations (*d* = -0.03). Except for Supportive social relations (*p* < 0.01), significances were observed at the *p* < 0.001-level.

Most of the contrasts between those who reported not to have been injured during the last 12 months and the “Injured twice or more”-group were medium large with *d*-values ranging from 0.34 to 0.54. Again, the exceptions were Positive social relations (*d* = -0.17) and Belongingness (*d* = -0.19). The strongest differences were found for Sleeping problems (*d* = 0.52), Global subjective health (*d* = 0.54), Distress (*d* = 0.52), Total quality of life (*d* = -0,47), and Negative affect (*d* = 0.46).

In Table 2, all outcome variables are analyzed against being injured during the last 12 months or not and cause of injury. One group deviate strongly from the reference group (reported not to have been injured during the last 12 months), namely those who reported to have been victims of violence. The *d*-values range between 0.61 and 1.01, which means the differences are from medium to large. There are three exceptions with less large differences: Positive social relations (*d* = -0.30), global subjective health (*d* = -0.44) and belongingness (*d* = -0.46). The largest differences are found for Feeling safe (*d* = -1.01), Distress (*d* = 0.92), Social capital (*d* = -0.86), Total quality of life (*d* = -0.78), and Sleeping problems (*d* = 0.74).

The differences between the mean scores of the reference group and the two other groups who reported to have been injured during the last 12 months (due to accidents, and “other cause”) were rather small.

Table 3 shows all outcome variables by injury-related daily life functional impairment. If reported to have been injured (not restricted to last 12 months), but with no or only small effects in daily life, differences with the reference group were small. Those who reported to have been injured, and with “some” impact on daily life, were more different from the reference category with most *d*-values in the range from 0.15 to 0.36. For Global subjective health the *d*-value was much higher, 0.71.

Those who reported to have been injured, and with strong impact on their daily life, d-values ranged from 0.35 to 1.17. Highest deviations from the reference groups were observed for Global subjective health (d = -1.17), Global quality of life (d = -0.95), Sleeping problems (d = 0.88), Total quality of life (d = -0.84), and Distress (d = 0.83).

### Missing data

The proportion of missing observations on outcome variables varied between less than 0.1% and 0.3%. The proportion of missing observations on predictors varied between 0.1% and 0.4%. Since information about gender and age was retrieved from the Norwegian National Population Register, these variables had no missing.

## Discussion

The present study has shown that being injured during the last 12 months is associated with elevated mean scores on psychological distress, sleeping problems, and loneliness, and lower mean scores on global subjective health and a series of quality-of-life indicators, when compared with those who did not report to have been injured during this period. The injury or injuries had to be sufficiently serious to require treatment from a medical doctor or a dentist. Being injured only once was only weakly associated with the outcome variables, while having been injured twice or more showed stronger associations with outcomes.

Those who had been victims to violence had on average much stronger deviations from the reference group than those who reported that their injury was caused by an accident.

Functional impairment due to injuries (not limited to last 12 months) with an impact on daily life functioning was associated with elevated mean scores on psychological distress, sleeping problems, and loneliness, and lower mean scores on global subjective health and a series of quality-of-life indicators, when compared with the reference group. Mean scores were particularly unfavourable among those with impairments which had strong effects on daily life functioning.

The present study seems to be the first one to examine relationships between being injured and a broad range of quality-of-life indicators and psychological distress based on a large community sample. Two Nordic studies [34, 35], have used a similar approach and found strong associations between being victims to violence and quality of life measures. The focus of these studies was, however, violence, and not injuries caused by violence. The validity of causal inferences with this kind of design rests on the unlikely assumption that those who were injured were not systematically different from the non-injured on outcome variables before injuries took place. This is also an obvious shortcoming of the present study.

An Australian study examined the combined effects on health-related quality of life of being victim to violence and being injured. Being injured was, however, in this study, measured independently of the measurement of being victim to violence [38]. Still it is interesting to note that the combination of having been injured and victim to violence was particularly strongly associated with low scores on the mental health component of health-related quality of life when measured with the 36-item Short-Form Health Survey (SF-36)[38].

We studied the association between being injured and distress and quality of life outcomes by comparing injured and non-injured people in a large community sample. However, other approaches exist in the literature. Some studies compare scores related to quality of life and distress among injured patients with similar scores based on normative populations [7, 14–23]. Others are based on comparisons of post-injury scores on quality of life and distress indicators with pre-injury (baseline) scores obtained retrospectively [7, 8, 10, 12, 31]. None of these alternative designs are without limitations. In the first case, finding comparison data where the same instruments are used on relevant populations at approximately the same time, might prove to be difficult. In the second case, the approach based on the assumption that data based on retrospective reports produces results close to what would have been obtained with pre-injury data collections is problematic. Comparisons of post-injury scores with pre-injury scores obtained retrospectively may be misleading. Social desirability [39] and “response shift” processes [40] may compromise the comparability of post-injury- with pre-injury measurements. Scholten and associates, in a review of studies, concluded that retrospective measurement of pre-injury health-related quality of life consistently produces higher pre-injury scores than population norms [30].

We have distinguished between three different approaches to studying associations between being injured and distress as well as quality of life outcomes: (i) comparisons of injured and non-injured in large community samples (such as in the present study), (ii) comparisons of injured patients with reference groups, and (iii) comparisons of injured patients’ post-injury scores with pre-injury scores with the latter being measured retrospectively. A high level of consistency in findings across the three approaches, medium to strong associations, clear dose–response-relationships, and the finding that scores on outcomes gradually approach pre-injury levels over time after the injury, contribute to some confidence in the findings of the present study. With regard to prospective, longitudinal studies, and consistent with what has been suggested by Scholten and associates, [30], we would maintain that obtaining baseline scores on health-related quality of life and distress prospectively instead of retrospectively would improve these studies considerably. To obtain this kind of study design, it would be necessary to have access to data from large community-based data collections, identify those who were injured during a specific time period after measurement, and carry out one or more post-injury data collections among injured as well as among non-injured.

Many studies have shown that being injured is associated with higher levels of distress and lower levels of health-related quality of life. The observation from previous research that quality of life improves and distress decreases as a function of time after the injury event [26, 32], adds to our conviction that being injured has negative psychological consequences. The present study has also confirmed that there are clear dose–response relationships (number of injuries, degree of functional impairment). It would, in fact, be surprising if a causal relationship between being injured and psychological outcomes did not exist. The exact strength and duration of effects may be more difficult to determine. In the context of previous research, with variations in research designs, we do, however, have strong indications that being injured leads to reduced quality of life and increased levels of psychological distress. In order to estimate the total economic and human costs of injuries, mental health and quality of life-related effects should be included in the calculations.

The present study was based on large community samples and well-tested instruments for data collections. There was, however, room for improvements regarding the measurement of injuries. There could have been questions on what part or parts of the body were injured, when the injury took place, how long time it took until full recovery, aspects of treatments, the nature of impairments associated with the injury, and the degree of dependency on helpers (health, personnel, family, others). Furthermore, we could have moved beyond use of cross-sectional data and collected data repeatedly in the same sample of study participants. With this kind of panel data, we could have adjusted for outcome variables which were also measured at time 1, and thereby examined prediction of change. And with a sufficient number of measurement occasions, it would be possible to utilize growth curve and cross-lagged models as well as more recent statistical tools for the analysis of interdependencies across time [50, 51]. This would be possible in future research by employing extended data collections in the context of the Norwegian Counties Public Health Survey.

### Strengths and limitations

The proportion who reported to have been injured seriously enough to see a medical doctor or a dentist during the last 12 months (17.6%) is somewhat higher in the present data than estimates based on register data [52, 53]. Perfect correspondence between estimates based on survey data and estimates based on register data cannot be expected. Some over-reporting is not, however, likely to noticeably bias the specific associations observed in this study.

In the data collections carried out under the umbrella of the Norwegian Counties Public Health Survey, we have experienced elevated levels of non-participation among elderly women, young men, those with the lowest level of completed education, and among immigrants from low- and middle-income countries. This kind of selection may contribute to reduced variability and have some consequences when data are used for descriptive purposes, but is unlikely to have much impact on direction and strength of associations. Reported in a previous publication based on data from one Norwegian county, analyses of differences between those who responded after first contact and those who responded after one or two reminders, revealed no systematic differences in outcomes between these three groups [54].

The scales and questions included in this study have not been tested for equivalence between the different modes of administration (smartphone, tablet, computers), and no information is available with regard to how the number of respondents was distributed across these modes.

Strengths of the present study includes a broad selection of outcome variables measured with well tested instruments, large community-based samples, and more detailed exposures including being injured twice or more, cause of injury, and degree of functional impairment due to injuries. Since the present study is based on cross-sectional data, causality inferences cannot easily be made. In order to have stronger evidence that injuries actually cause reduced quality of life and increased distress, we would have to adjust for outcome variables measured before the injury event(s) took place.

## Data Availability

Data are securely stored at a server belonging to the Norwegian Institute of Public Health. Data from the Norwegian Counties Public Health Surveys are available to researchers who have received approval from the Norwegian Institute of Public Health and relevant ethical committees.
